# Long-term continuous cultivation of Kenyan infant fecal microbiota using the host adapted PolyFermS model

**DOI:** 10.21203/rs.3.rs-3101157/v1

**Published:** 2023-07-07

**Authors:** Carole Rachmühl, Christophe Lacroix, Paula Momo Cabrera, Annelies Geirnaert

**Affiliations:** ETH Zurich; ETH Zurich; ETH Zurich; ETH Zurich

## Abstract

Appropriate *in vitro* models to investigate the impact of novel nutritional strategies on the gut microbiota of infants living in rural Africa are scarce. Here, we aimed to develop such a continuous gut fermentation model based on the PolyFermS platform.

Eight immobilized Kenyan infant fecal microbiota were used as inoculum for continuous PolyFermS colon models fed with medium mimicking the weaning infant diet. Fructo-oligosaccharides (FOS) supplementation (1, 4 and 8 g/L) and cultivation pH (5.8 and 6.3) were stepwise investigated. Conditions providing a close match between fecal and *in vitro* microbiota (pH 5.8 with 1 g/L FOS) were selected for investigating long-term stability of four Kenyan infant PolyFermS microbiota.

The shared fraction of top bacterial genera between fecal and *in vitro* microbiota was high (74–89%) and stable during 107 days of continuous cultivation. Community diversity was maintained, and two distinct fermentation metabolite profiles, propiogenic and butyrogenic, of infant fecal microbiota established from day 8 onwards and stayed stable.

We present here the first rationally designed and accurate continuous cultivation model of African infant gut microbiota. This model will be important to assess the effect of dietary or environmental factors on the gut microbiota of African infants with high enteropathogen exposure.

## Introduction

*In vitro* gut fermentation models allow to study the impact of dietary factors on the gut microbiota community and functionality independent of the host. Development of gut fermentation models requires careful selection and adjustment of the operation parameters to closely mimic the target host intestinal environment [1]. Batch fermentation models are most frequently used to screen dietary compounds but are limited to short-time experiments (48–72 h) [2, 3]. For long-term in-depth gut microbiota studies, continuous fermentation models are used to better mimic the host gut environment and colon specific parameters and to allow measurement of factors in stable conditions.

To date, only one study reported the use of a semi-continuous fermentation model for undernourished Burkina-Faso infants using the baby-SHIME model inoculated with infant feces previously frozen without cryoprotectant [4]. A cultivation medium similar to the baby-SHIME feed, which is based on commercial infant formula, but with 20% lower amounts of nutrients to mimic malnutrition status was used. The validity of *in vitro* gut fermentation models is however highly dependent on the handling and preservation of the fecal donor community and activity, and on the selected cultivation conditions [1]. Freezing and lack of close adjustment to the host diet can add significant bias to a model by affecting fecal microbiota viability. Further, the *in vitro* gut microbiota composition and activity must be compared to the donor fecal microbiota to demonstrate the representativity and validity of the model, which has not been done before for infants living in low- and middle-income countries (LMIC) and under low hygiene conditions. A high enteropathogen prevalence is frequenctly detected in these infants and is an important characteristic to be reproduced for reliable *in vitro* studies [5–8].

A first attempt has been made to mimic *in vitro* the gut microbiota of LMIC infants from rural Kenya using the continuous PolyFermS model [9]. However, Swiss infant fecal microbiota used for cultivation in conditions selected to mimic the colon and the infant diet during weaning. Artificial contamination of the cultivated infant fecal microbiota with enteropathogens further allowed to approximate the natural high pathogen exposure of the Kenyan infant gut. However, such an approach lacks the native target host microbial species of the native infant microbiota. The gut microbiota composition of LMIC infants is indeed different from the gut microbiota from infants living in high-income countries [10, 11]. We recently demonstrated preservation of the community and activity of fresh Kenyan infant fecal microbiota after cooled anaerobic transport for batch cultivations in host-diet adapted conditions and with inoculation within less than 30 h after sample collection [12]. A high fraction (98 ± 5%) of top bacterial genera (≥ 1% abundant) was shared between fecal and *in vitro* cultivated microbiota and metabolic activity was shown to be well-reproduced *in vitro*.

The aim of this study was therefore to select conditions closely mimicking *in vitro* the gut microbial community of LMIC infants in the continuous PolyFermS fermentation model. PolyFermS models were inoculated with immobilized fresh fecal microbiota of Kenyan infants living in low hygiene environment. The fermentation medium composition was adapted to mimic the daily Kenyan infant chyme entering the colon, with a diet consisting of human milk and complemented with porridge, fruits and vegetables and the physiological conditions of the colon of 6–8 months old African infants. First, different amounts of fructo-oligosaccharides (FOS) were assessed to promote *Bifidobacterium* growth, which is characteristic for the infant gut microbiota. Next, different cultivation pH were tested to support the growth of potential enteropathogens, which are commonly detected in feces of this infant population [5]. Finally, community stability in the model effluent was assessed during long-term continuous cultivation of four different Kenyan infant fecal microbiota in the adjusted PolyFermS model.

## Results

### Composition and metabolite profile of Kenyan infant donor fecal microbiota

**Figure 1:**
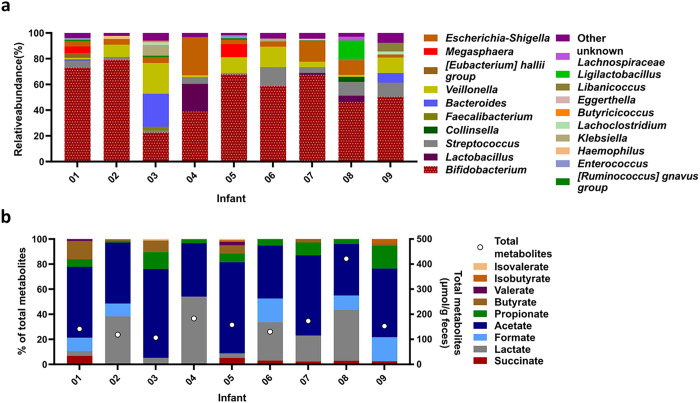
Microbiota composition and metabolite profile of Kenyan infant fecal microbiota measured with 16S rRNA gene amplicon sequencing and HPLC analysis. Relative abundance of dominant fecal genera (≥1%), “other” are the summed genera with abundance <1% (**a**). Relative proportion of fecal metabolites (left y-axis) and total fecal metabolite concentration (right y-axis, open circles) (**b**).

Freshly collected fecal samples from 9 Kenyan infants were transported under anaerobic and cooled conditions from Kenya to Switzerland for processing, immobilization and reactor inoculation within 28 to 35 h after collection [12]. The dominant genus in all fecal microbiota was *Bifidobacterium* (60 ± 14%) except for fecal microbiota of infant 03, which was dominated by *Bacteroides* (26%), Veillonella (24%) and *Bifidobacterium* (23%) (Fig. 1a). Total fecal metabolites differed between the infants with concentrations ranging between 106 μmol/g feces (infant 3) and 421 μmol/g feces (infant 08) (Fig. 1b). Acetate was the most abundant fecal metabolite (41 to 73% of total metabolites). A high variability in detection and proportions of propionate (2 to 18%), butyrate (2 to 15%), formate (10 to 19%) and lactate (4 to 54%) was observed. The proportions of valerate and BCFA were low in the fecal samples of all infants when detected (ranging from 1 to 3% of total metabolites).

In summary, the Kenyan infant donor fecal microbiota was characterized by high proportions of *Bifidobacterium* and acetate, while the proportions and presence of other taxa and metabolites was variable. The accordance with results obtained by previous studies in the same infant population confirms the representativity of the fecal samples used in this study to assess the optimal conditions for the continuous cultivation in the PolyFermS model [5, 12].

### Effect of different FOS doses on PolyFermS Kenyan infant fecal microbiota

**Figure 2:**
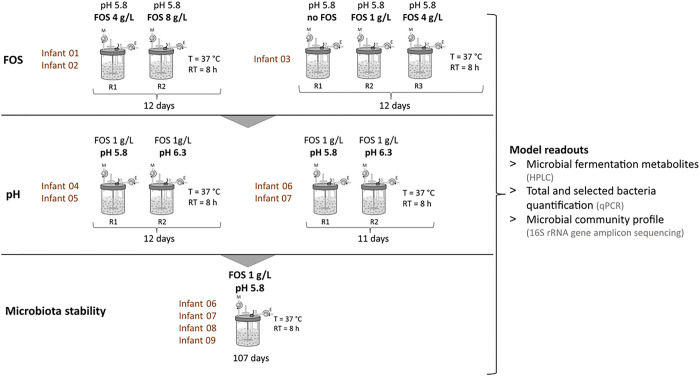
Experimental set up of PolyFermS continuous fermentation during model parameter adjustment and long-term microbiota stability assessment. The fecal microbiota of each infant was immobilized separately and cultivated in individual reactors (R), with continuous supply of nutritive medium (M) and removal of effluent (E). Tested operation parameters and cultivation duration in days are indicated. Final FOS concentrations in the nutritive medium are shown. T = temperature, RT = retention time, PCR = polymerase chain reaction, HPLC = high performance liquid chromatography

Different concentrations of FOS were added to the cultivation medium, simulating the Kenyan infant diet and ileal chyme. The objective was to determine wether FOS could promote the *in vitro* growth of *Bifidobacterium*. This assessment was conducted using three independent PolyFermS models, where each bioreactor contained immobilized fecal microbiota of one Kenyan infant (Fig. 2). Composition and metabolite profile of the bioreactor effluent *in vitro* microbiota (IVM) were compared to the respective fecal inoculum.

### Quantitative PCR enumeration of key bacterial taxa

IVM 01 and 02 were cultivated with 4 g/L and 8 g/L of FOS and IVM 03 was tested without supplementation and with 1 g/L and 4 g/L of FOS. Compared to the corresponding fecal inoculum, lower concentrations of *Bifidobacterium* in reactors effluent were detected for all FOS doses tested (**Supplementary table 1**). The concentrations of *Bifidobacterium* were lowest with 8 g/L of FOS and 0.99 and 0.60 log lower compared to 4 g/L of FOS for IVM 01 and 02, respectively. Concomitant, growth stimulation of *Ruminococcaceae* was observed with 8 g/L FOS compared to 4 g/L and to much higher concentrations than detected in the fecal microbiota. Therefore, in a next step, lower FOS doses of 1 g/L and 4 g/L were assessed for IVM 03. FOS supplementation did not impact *Bifidobacterium* concentrations in IVM 03, but higher concentrations of *Ruminococcaceae* were measured with 4 g/L FOS compared to the non-supplemented condition. FOS-induced infant-specific changes in key bacterial taxa were also detected. Concentrations of *Lachnospiraceae, Enterobacteriaceae* and *Bacteroides* were decreased in IVM 01 and *Lachnospiraceae* concentrations were lower in IVM 02 with 8 g/L compared to 4 g/L of FOS. Increased concentrations of *Lachnospiraceae*, *Veillonella* and *Lactobacillus /Leuconostoc/Pediococcus (LLP)* were detected in IVM 03 with 4 g/L of FOS compared to non-supplemented condition (**Supplementary table 1**).

In summary, FOS supplementation stimulated the growth of *Ruminococcaceae* in all IVM while *Bifidobacterium* decreased in IVM 01 and 02 supplemented with 8 g/L of FOS compared to 4 g/L.

### Sequencing-based microbial community profile

**Figure 3:**
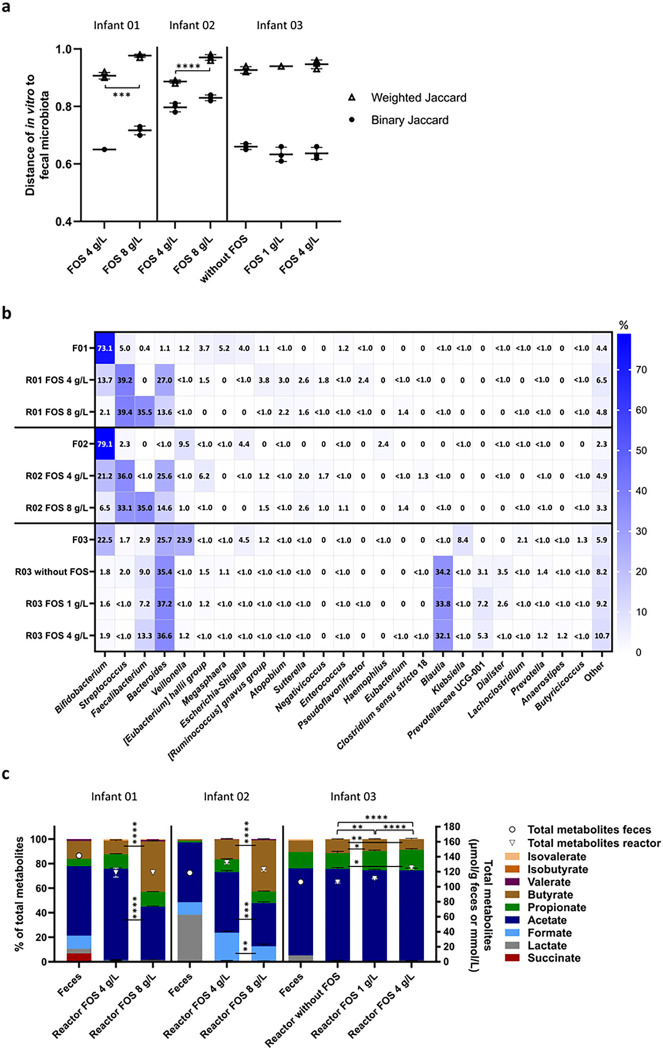
Composition and metabolite profile of fecal inoculum and *in vitro* Kenyan infant fecal microbiota exposed to different doses of FOS measured with 16S rRNA gene amplicon sequencing and HPLC. Binary and weighted Jaccard distance between fecal and *in vitro* microbiota (a). Distance calculations were done separately for each infant microbiota. Distance of 0 indicates identical communities while 1 indicates different communities. Relative abundance of abundant bacterial genera (≥1%) detected in fecal and *in vitro* microbiota, “other” are the summed genera with abundance <1% (**b**). F denotes feces, R reactor (*in vitro*). Relative proportion of SCFA, BCFA and intermediate metabolites (left y-axis) and total metabolite concentration (right y-axis, open symbols) (**c**). Significant differences in the proportions of metabolites are indicated between the bars and significant differences in total metabolite concentrations above the bars. Mean (±SD for **a** and **c**) of the last three days of fermentation is shown. Mean of technical duplicates is shown for feces. *p<0.05, **p<0.01, ***p<0.001, ****p<0.0001

Structural and compositional similarity of fecal and *in vitro* microbiota were evaluated with beta diversity metrics. The similarity between both was significantly lower for IVM 01 and 02 cultivated with 8 g/L of FOS compared to 4 g/L based on weighted Jaccard similarity index while no differences were observed for IVM 03 (Fig. 3a).

In line with quantitative PCR (qPCR) analysis, the relative abundance of *Bifidobacterium* was lower *in vitro* compared to the fecal inoculum microbiota in all three IVM (Fig. 3b). In IVM 01 and 02, supplementation with 8 g/L compared to 4 g/L of FOS, promoted *Faecalibacterium* (35.5% and 35.0% relative abundance versus 0 and < 0.1%, respectively) at the expense of *Bacteroides* (13.6% and 14.6% versus 27% and 25.6%, respectively) and *Bifidobacterium* (2.1% and 6.5% versus 13.7% and 21.2%, respectively). In IVM 03, the relative abundance of *Bifidobacterium* was low (1.6 to 1.9%) independent of FOS treatment, while the relative abundance of *Faecalibacterium* was also increased with FOS supplementation (13.2% with 4 g/L, 7.1% with 1 g/L and 8.7% without FOS). Several bacterial genera harbouring potential pathogens were detected in IVM effluents but at a low er relative abundance compared to the respective fecal inoculum. For example, the relative abundance of *Escherichia-Shigella* was below 1% (0 to 0.6%) in all IVM, while it ranged from 4.0–4.5% in the fecal microbiota. Also, *Klebsiella* was detected at a relative abundance of 8.4% in fecal microbiota of infant 03 but only at 0.1–0.4% in IVM 03.

Differential abundance analysis confirmed the promotion of *Faecalibacterium* with increasing FOS doses *in vitro* (**Supplementary Fig. 1**). *Faecalibacterium* (ASV 037 assigned to F. prausnitzii) showed an increase in relative abundance with 8 g/L versus 4 g/L of FOS in IVM 01 (log2-fold increase 16.9) and IVM 02 (log2-fold increase 13.9), and with 1 g/L versus no FOS and 4 g/L versus 1 g/L of FOS in IVM 03 (log2-fold increase 1). Concomitant, a decreased relative abundance of *Bifidobacterium* was detected with 8 g/L versus 4 g/L of FOS in IVM 01 (log2-fold decrease − 2.3) and IVM 02 (log2-fold decrease − 1.2).

Increasing FOS doses also impacted alpha diversity *in vitro*. The community richness of IVM 01 was lower at 8 g/L compared to 4 g/L of FOS and compared to the corresponding fecal microbiota (**Supplementary Fig. 2**). Furthermore, the community evenness of IVM 01 and 02 was higher compared to the corresponding fecal microbiota and increasing FOS doses reduced the evenness in all tested IVM, which might be explained by the strong promotion of *Faecalibacterium*.

### Fermentation metabolite profile

Compared to the fecal metabolite profile, the ratio of intermediate to total metabolites decreased, and that of propionate and butyrate increased in IVM 01 and 02 (Fig. 3c). Butyrate production was largely enhanced in IVM 01 and 02 with 8 g/L of FOS compared to 4 g/L, representing 41% and 42% versus 11% and 16% of total metabolites, respectively, and at the expense of acetate. The metabolite profile of IVM 03 was very similar compared to the fecal metabolite profile, independent of the FOS treatment, except for lactate which was only detected in the feces.

Overall, *Bifidobacterium* and genera harbouring potential pathogens were maintained *in vitro* but at lower abundance compared to the fecal microbiota. With the high FOS doses of 8 g/L and 4 g/L the relative abundance of *Bifidobacterium* decreased concomitant with increased *Faecalibacterium* and largely enhanced butyrate production, compared to the fecal microbiota. Therefore, FOS at 1 g/L, was selected for reproducing a representative profile of the Kenyan infant fecal microbiota in the *in vitro* PolyFermS fermentation model.

#### Effect of pH on in vitro establishment of Kenyan infant fecal microbiota

The effect of an increased cultivation pH from 5.8 to 6.3 was tested in four continuous fermentation models inoculated with fecal microbiota from infant 04 to 07 to assess if a higher cultivation pH supports the *in vitro* establishment of potential enteropathogens present in Kenyan infant feces.

### qPCR enumeration of key bacterial taxa

Cultivation pH had a donor-dependent impact on the growth of *Enterobacteriaceae*, a bacterial family that harbours many potential pathogenic genera including *Escherichia, Shigella* and *Salmonella*.

*Enterobacteriaceae* concentrations were 1.6 log higher at pH 6.3 compared to pH 5.8 in IVM 04, while they were 0.5 and 0.8 log lower in IVM 06 and 07, respectively (**Supplementary table 2**). A pH of 6.3 promoted the growth of *Bacteroides* in IVM 04 (+ 0.5 log) and IVM 06 (+ 2.6 log), and of *Lachnospiraceae* in IVM 04 (+ 0.8 log) and IVM 05 (+ 0.5 log) compared to pH 5.8. Moreover, higher cultivation pH 6.3 decreased the concentrations of infant-characteristic *Lactobacillus /Leuconostoc/Pediococcus* in IVM 04 to 07 (−0.5 to −2.2 log) and *Bifidobacterium* in IVM 04 to 06 (−0.1 to −1.2 log) compared to pH 5.8. *Veillonella* concentrations were also 1.7 log lower at pH 6.3 compared to 5.8 in IVM 06.

### Sequencing-based microbial community profile

**Figure 4:**
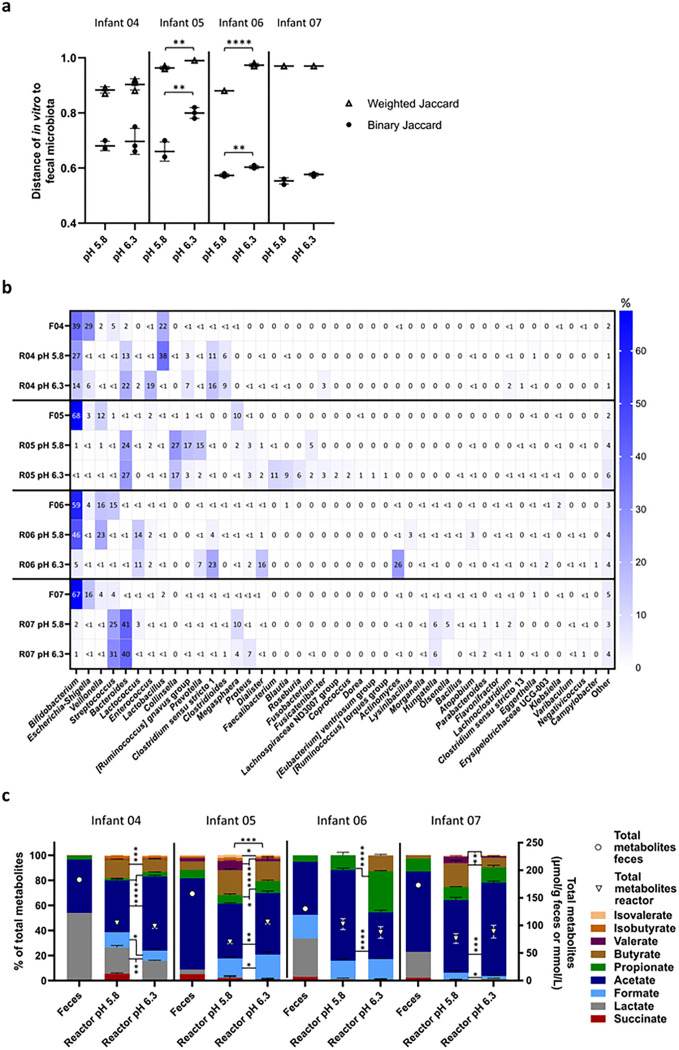
Composition and metabolite profile of fecal inoculum and *in vitro* Kenyan infant fecal microbiota cultivated at different pH measured with 16S rRNA gene amplicon sequencing and HPLC. Binary and weighted Jaccard distance between fecal and *in vitro* microbiota (**a**). Distance calculations were done separately for each infant microbiota. Distance of 0 indicates identical communities while 1 indicates different communities. Relative abundance of dominant bacterial genera (≥1%) detected in fecal and *in vitro* microbiota, “other” are the summed genera with abundance <1% (**b**).F denotes feces, R reactor (*in vitro*). Relative proportion of SCFA, BCFA and intermediate metabolites (left y-axis) and total metabolite concentration (right y-axis, open symbols) (**c**). Significant differences in the proportions of metabolites are indicated between the bars and significant differences in total metabolite concentrations above the bars. Mean (±SD for **a**and **c**) of the last three days of fermentation is shown. Mean of technical duplicates is shown for feces. F denotes feces, R reactor (*in vitro*). *p<0.05, **p<0.01, ***p<0.001, ****p<0.0001

The community distance of IVM 05 and IVM 06 to the corresponding fecal microbiota was higher after cultivation at pH 6.3 compared to pH 5.8 (Fig. 4a). Infant-specific and common compositional shifts in relative genera abundance were detected after cultivation (Fig. 4b). The relative abundance of *Escherichia-Shigella* was low in all IVM (0.02–6.32%) compared to the fecal microbiota (3.3–29.1%) and an enrichment of *Bacteroides* (12.6–38.5% in IVM versus 0.2–2.0% in feces) was observed in IVM 04, 05 and 07. Increased cultivation pH resulted in several infant-specific shifts in relative genus abundance. For example, in IVM 04 at pH 6.3 compared to 5.8, the relative abundance of *Enterococcus* was increased (18.9% at pH 6.3 versus 0.2% at pH 5.8, respectively) at the expense of *Lactobacillus* (0.2% versus 37.7%, respectively). In IVM 05, the relative abundance of *Faecalibacterium* (11% versus 0%, respectively) and *Blautia* (9% versus 0%, respectively) was increased while the relative abundance of *Prevotella* (2% versus 15%, respectively) was decreased at pH 6.3 compared to 5.8. In IVM 06, the strongest increase in relative abundance at pH 6.3 compared to 5.8 was observed for *Clostridium* sensu stricto 1 (23% versus 4%, respectively), *Dialister* (16% versus 1%, respectively) and *Actinomyces* (26% versus 0%, respectively). Furthermore, at pH 6.3, the relative abundance of *Bifidobacterium* was strongly decreased to 0.2% and 3.9% in IVM 04 and 06, respectively, compared to pH 5.8 where it was among the three most abundant genera (27% and 37%, respectively). In contrast, independent of cultivation pH, *Bifidobacterium* relative abundance was low in IVM 05 and 07 (0.2–2.1%).

Differential abundance analysis was performed to detect pH-induced significant differences in relative genus abundance. Higher cultivation pH 6.3 promoted genera harbouring potential pathogenic gut members (**Supplementary Fig. 3**). The relative abundance of *Escherichia-Shigella* increased at pH 6.3 compared to 5.8 for IVM 04, 05 and 06 with a log2-fold increase from 3.0 to 3.9. *Clostridium* sensu stricto 1 was also promoted at pH 6.3 as indicated by a log2-fold increase of 7.3 and 2.9 compared to pH 5.8 in IVM 05 and 06, respectively. Moreover, at pH 6.3 compared to pH 5.8, a log2-fold increase of 10.9 and 3.2 was detected for *Campylobacter* and *Clostridioides* in IVM 06 and 07, respectively. On the other hand, and in line with qPCR results, cultivation pH 6.3 decreased the relative abundance of infant-characteristic beneficial gut taxa such as *Bifidobacterium* in IVM 04, 06 and 07 and *Lactobacillus* in IVM 04 and 06, respectively, at pH 6.3 compared to 5.8.

Alpha diversity was also affected by cultivation pH, with a much higher number of observed ASVs at pH 6.3 (85 ± 4 ASVs) compared to 5.8 (49 ± 6 ASVs) in IVM 05 and compared to its fecal inoculum (38 ASVs) (**Supplementary Fig. 4**). The community evenness of all IVM was higher compared to the corresponding fecal microbiota and cultivation at pH 6.3 compared to 5.8 further increased the evenness in IVM 04 and 06.

### Fermentation metabolite profile

Total metabolite concentrations were lower in all IVM compared to the fecal microbiota (Fig. 4c). Compared to the fecal metabolite profile, proportions of intermediate metabolites were decreased concomitant with increased proportions of acetate, propionate and butyrate in all IVM except for IVM 05 (due to formate production *in vitro*). Cultivation pH 6.3 stimulated propionate production while pH 5.8 promoted butyrate and valerate production (except in IVM 06 where butyrate was not detected at pH 5.8). Large increase of propionate proportion (33 ± 1% at pH 6.3 versus 12 ± 2% at pH 5.8), concomitant with decreased acetate proportions were observed for IVM 06.

In summary, higher cultivation pH 6.3 stimulated propionate production and genera harbouring potential pathogenic gut members compared to pH 5.8. However, infant-characteristic *Bifidobacterium* and *Lactobacillus* were better maintained *in vitro* at pH 5.8 resulting in higher similarity between fecal and IVM compared to pH 6.3. Therefore, pH 5.8 was chosen for long-term continuous cultivation.

### Model stability during long-term continuous cultivation of four Kenyan infant fecal microbiota

Four Kenyan infant fecal microbiota were continuously cultivated for up to 107 days to assess the community stability at the selected conditions (pH 5.8 and 1g/L FOS).

### Sequencing-based microbial community profile

**Figure 5:**
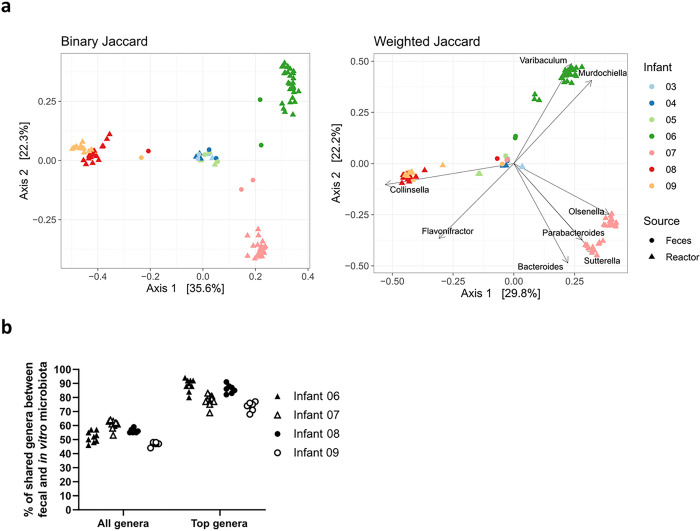
Beta diversity and shared genera of Kenyan infant fecal microbiota cultivated at pH 5.8 with 1 g/L of FOS. PCoA of binary and weighted Jaccard distance of feces and *in vitro* (reactor) microbiota 03 to 09 including all available fermentation days (**a**). *In vitro* microbiota 03 to 05 were included as additional reference. Genera significantly associated with the community structure are plotted as vectors in weighted Jaccard PCoA. Percentage of shared genera between feces and *in vitro*microbiota of infant 06 to 09 considering all genera or most abundant (≥1%) genera over the course of fermentation (**b**). Data points represent the mean of three consecutive fermentation days.

Principal coordinate analysis (PCoA) was conducted to assess whether the infant-specific fecal microbiota profile was maintained *in vitro* during cultivation at pH 5.8 with 1 g/L of FOS (Fig. 5a). PCoA of binary Jaccard showed clustering of *in vitro* microbial communities by infant. However, in the PCoA of weighted Jaccard, all the fecal microbial communities clustered together, which is likely due to similar high relative abundance of *Bifidobacterium* in contrast to the IVM. The percentage of shared genera between feces and IVM was stable over 107 days of continuous cultivation at 52 ± 5%, 61 ± 4%, 56 ± 3% and 47 ± 3% for IVM 06, 07, 08 and 09, respectively (Fig. 5b). When only the most abundant genera (≥ 1%) were considered, the percentage of shared genera between feces and IVM was higher at 89 ± 5%, 78 ± 5%, 86 ± 4% and 74 ± 4% for IVM 06, 07, 08 and 09, respectively.

**Figure 6:**
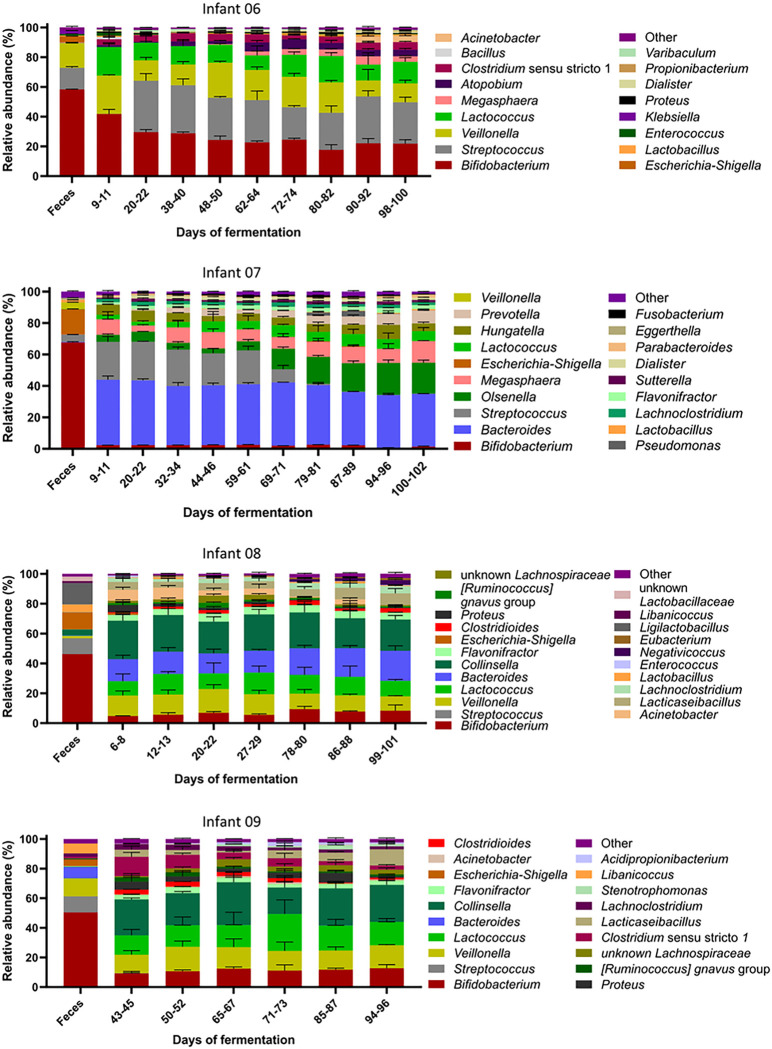
Composition of four Kenyan infant fecal microbiota during long-term continuous cultivation at pH 5.8 with 1 g/L of FOS measured with 16S rRNA gene amplicon sequencing. Relative abundance of abundant bacterial genera (≥1%) detected in feces and *in vitro* microbiota over the course of fermentation, “other” are the summed genera with abundance <1%. Mean ± SD of three consecutive days of fermentation is shown. Mean of technical duplicates is shown for feces. Samples of *in vitro* microbiota 09 were only sequenced from day 43 onwards.

Compared to the fecal microbiota, *Lactococcus*, *Streptococcus, Bacteroides* and *Collinsella* were enriched *in vitro* (+ 8% to + 40% compared to feces) concomitant with a decrease in *Bifidobacterium* abundance (−28% to −65% compared to feces) (Fig. 6). However, the abundant genera of all IVM were maintained over time, except for *Olsenella* outcompeting *Streptococcus* in IVM 07. Further, the abundance of *Streptococcus* was high (28–35%) in IVM 06 from day 20 onwards compared to below 1% at day 9–11. The community richness decreased while the evenness increased in all IVM compared to the fecal inoculum (**Supplementary Fig. 5**). Alpha diversity was stable over the course of fermentation and similar between all IVM (observed ASVs: 46–55, Pielou’s evenness index: 0.60–0.66). Only IVM 06 showed a significant but small increase of evenness (0.07, p = 0.02) from day 9–11 to day 98–100.

### Fermentation metabolite profile

**Figure 7:**
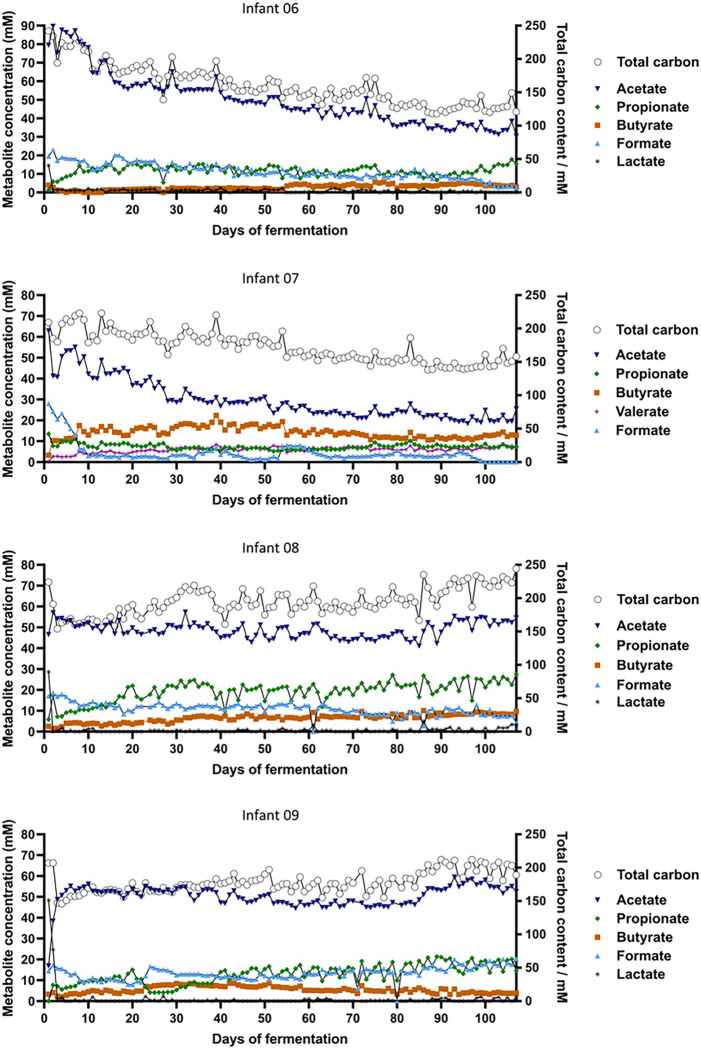
Metabolite profile of four Kenyan infant fecal microbiota during long-term continuous cultivation at pH 5.8 with 1 g/L of FOS measured with HPLC. Concentrations of intermediate fermentation metabolites and SCFA (left y-axis) and total carbon content (right y-axis, open circles) are shown over the course of fermentation. BCFA were below the detection limit.

The main metabolites detected in all IVM were acetate, followed by propionate, formate and butyrate (Fig. 7). After the initial 2 days of continuous fermentation, the intermediate metabolite lactate decreased to very low or non-detectable levels in all IVM. IVM 06, 08 and 09 established a propiogenic metabolite profile characterized by main SCFA ratio (acetate:propionate:butyrate) of 76:19:5, 66:26:8 and 74:18:8, respectively, in agreement with the propiogenic fecal metabolite profile (Fig. 3). In contrast, IVM 07 developed a butyrogenic profile with a main SCFA ratio of 57:15:28, while the fecal metabolite profile was propiogenic (83:13:4). The metabolite profiles were maintained over the complete cultivation period in all IVM, except for IVM 09 where a decrease in propionate between day 23 and 33 was observed followed by a recovery. Total metabolic activity over time was assessed considering total carbon concentrations to account for different carbon content of fermentation metabolites. The coefficient of variation over the complete fermentation time was comparable between IVM with 19%, 13%, 11% and 14% for IVM 06, 07, 08 and 09, respectively.

In summary, the Kenyan infant PolyFermS model maintained the most abundant genera, community diversity and specific metabolite profiles in IVM effluents over up to 107 days continuous fermentation.

## Discussion

Continuous fermentation models enable *in vitro* cultivation of gut microbiota in conditions reflecting the host gut environment [1]. The continuous PolyFermS model inoculated with immobilized fecal microbiota produces stable communities at high cell densities and with both sessile and planktonic bacterial populations as found in the gut [13, 14]. PolyFermS models were successfully applied for mimicking the colonic microbiota of different age human, from 2 months to 70 years [9, 13, 15]. In this study we present the adjustment of the PolyFermS model to closely mimic the gut microbiota of infants living in low hygiene conditions in rural Kenya. Different factors were stepwise investigated, including FOS supplementation (1, 4 and 8 g/L) and cultivation pH (5.8 and 6.3). Then, conditions providing a close match between infant feces and *in vitro* reactor microbiota composition were selected for investigating the stability of the PolyFermS model over up to 107 days continuous cultivation of four Kenyan infant fecal microbiota.

FOS was supplemented to support the growth of the infant-characteristic genus *Bifidobacterium*, which was previously low during continuous fermentation of Swiss infant fecal microbiota [9]. Increasing FOS dose stimulated the growth of *Faecalibacterium* in three Kenyan infant fecal microbiota *in vitro*. In contrast to the study hypothesis, the high FOS dose of 8 g/L resulted in a decrease in, while it significantly enhanced butyrate production compared to 4 g/L. Competition between both *Faecalibacterium* and *Bifidobacterium* taxa might explain these results. A FOS-induced increase in *Faecalibacterium* has not been reported so far in infant fecal microbiota cultivation studies [16–18], but was observed previously in single culture experiments via direct substrate consumption or in co-culture through cross-feeding of FOS-degrading and acetate-producing *Bifidobacterium* species to acetate-consuming and butyrate-producing *Faecalibacterium prausnitzii* [19–21]. The relative abundance of *Faecalibacterium* was shown to increase in both the fecal microbiota of Western and Kenyan infants during weaning between the age of 6 to 12 months and ranged between 1 to 10% [22–24]. Therefore, the relative abundance of *Faecalibacterium* at 7% as observed in this study for IVM 03 with the FOS dose of 1 g/L is representative for *Faecalibacterium* at this infant age [25].

Genera harbouring potential pathogenic gut members were decreased compared to feces during continuous fermentation at pH 5.8. Higher cultivation pH 6.3 stimulated the growth of these genera but decreased the abundance of infant fecal microbiota-dominant *Bifidobacterium* and *Lactobacillus* compared to pH 5.8. Potential pathogenic gut members, such as *Escherichia coli* and *Campylobacter,* are known to grow optimal around pH 6.5–7.0 [26, 27], while the pH-tolerance of *Bifidobacterium* and *Lactobacillus* [28, 29] confers a competitive advantage at lower cultivation pH and might explain the increased abundance observed after cultivation at pH 5.8 compared to 6.3. The overall higher microbial community similarity of the Kenyan infant fecal and *in vitro* microbiota cultivated at lower pH 5.8 compared to pH 6.3, and the previously reported fecal pH measurements ranging between 5.3 to 5.7 [5] [30] [6] in the same study population suggest that a cultivation pH below 6.0 reflects better the *in vivo* colon condition.

The adjusted PolyFermS model successfully maintained the most abundant genera, including *Bifidobacterium*, as well as the community diversity, and specific metabolite profiles over up to 107 days continuous fermentation of four Kenyan infant fecal microbiota at pH 5.8 and 1 g/L of FOS. Compared to the fecal microbiota, *Bifidobacterium* relative abundance was decreased while *Lactococcus*, Streptococcus, *Bacteroides* and *Collinsella* were enriched *in vitro*. However, compositional shifts are expected *in vitro* as the fecal microbiota is only a proxy of the colon microbiota [31]. Moreover, the observed *in vitro* establishment of not only propiogenic but also butyrogenic metabolite profiles is expected as butyrate production by the infant gut microbiota increases at the age of and during weaning [23, 24]. Besides maintaining infant-specific compositional and metabolite profiles, the diversity and metabolic activity of all fecal microbiota became more similar *in vitro*, which can be explained by exposure to the same fixed growth conditions in a well-controlled and reproducible experimental procedure.

We present here the first continuous cultivation of fecal microbiota from LMIC infants starting from an active fecal inoculum. To ensure validity, the model was inoculated with feces transported under protective conditions from infants living in rural Kenya and fed with medium designed to closely mimic the host diet [12]. Comparison of the *in vitro* gut microbiota composition and activity to the donor fecal microbiota further demonstrated the representativity of the model. This comparison was missing for the continuous fermentation model of undernourished Burkina-Faso infants that used previously frozen feces for cultivation in a medium lacking close adjustment to the host diet [4]. Moreover, compared to the previous PolyFermS model, which was inoculated with Swiss infant fecal microbiota [9], the use of native Kenyan infant fecal microbiota together with the supplementation of FOS and the longer retention time in the presented model allowed to closely mimic the Kenyan infant gut microbiota.

This is the first validated continuous fermentation model using active fecal microbiota from infants living in a rural area of Kenya which allowed to cultivate a representative community of the fecal microbiota over long-term. The Kenyan infant PolyFermS model can be used to study the direct effect of dietary compounds, drugs or antibiotics on the gut microbiota of infants living in rural sub-Saharan Africa where novel nutritional solutions are currently investigated to mitigate adverse effects of iron fortification on the gut microbiota [5, 22].

## Methods

### Donor characteristics

Fresh fecal samples were collected from 9 infants living in Msambweni County in southern coastal Kenya (Table 1). All infants, except of infant 03, did not receive antibiotics in the 12 weeks before sample donation. Infant 03 was treated with oral amoxicillin/penicillin and gentamicin ear drops 6 weeks before sampling. No illness was reported for the other infants. The average age of the infants was 9.0 ± 1.1 months. All infants were born vaginally and term (except for infant 03). No information was available about gestational age at birth for infant 01 and 02. The average birth weight was 2.8 ± 0.6 kg. Seven infants were mixed fed with human milk and complementary foods that consisted predominantly of a maize porridge called “uji” besides vegetables and fruits. The average time since the start of weaning was 3.5 ± 1.8 months. Infant 06 and 07 were still exclusively breast-fed. The average fecal pH was 5.2 ± 0.9 (ranging from 4.3 to 7.3).

### Fecal sample collection

Fecal samples were collected and transported under protective conditions as previously described [12]. In brief, the fresh fecal samples were aliquoted from diapers into sterile plastic tubes and immediately transferred to a gastight anaerobic jar containing an anaerobic atmosphere generating system and cold packs for cooling. The samples were air-transported from Kenya to Zurich for further processing, immobilization and reactor inoculation within 28 to 35 h after collection. The sample temperature measured at arrival in the laboratory ranged from 4 to 8°C. Fecal sample aliquots for DNA extraction and pH measurement were stored at −80°C until further use.

### Fecal pH measurement

pH was measured as previously described [12]. In brief, 1 mL of distilled water (dH_2_O) was added to 100–120 mg of thawed fecal sample and the solution was homogenized by vortexing prior to pH measurement with a pH electrode (Methrom, Zofingen, Switzerland).

### PolyFermS in vitro continuous colonic fermentation

#### Fecal bacteria immobilization and fermentation conditions

Fecal microbiota were immobilized anaerobically into 1–2 mm gel beads composed of gellan gum (2.5%), xanthan gum (0.25%) and sodium citrate (0.2%) using a two-phase dispersion process as previously described [32]. Freshly produced fecal beads (60 mL) were transferred into a bioreactor containing 140 mL of cultivation medium (30% (v/v), in 500 mL bioreactors from the Multifors system, Infors AG, Bottmingen, Switzerland, or DASbox system, Vaudaux-Eppendorf AG, Basel, Switzerland). To colonize the beads, two consecutive batch fermentations (20 and 6 h) were carried out by aseptically replacing 100 mL of fermented with fresh cultivation medium. After batch colonization, reactor operation was switched to continuous mode. Operation parameters were selected for mimicking the colon of 6 to 10 month old infants living in rural Africa. The flow rate was set to 25 mL/h for a mean retention time of 8 h reflecting the high stool frequency of 3 to 4 per day as reported in African infants with high pathogen exposure [33]. The pH was controlled at 5.8 (if not otherwise stated) by addition of 2.5 M NaOH and is within the range of detected fecal pH in the same Kenyan infant population [5]. All reactors were operated at 37°C and stirring at 120 rpm. The reactor headspace and medium bottles were continuously flushed with filter-sterile CO_2_ to ensure anaerobiosis.

### Cultivation medium

The cultivation medium was designed and validated to mimic the ileal chyme entering the colon of Kenyan infants during weaning at the age of 6 to 8 months as previously described [12]. The representative daily diet consists of 500 mL mother milk and 330 g uji, a maize or millet porridge, complemented with fruits and vegetables. The medium composition was as follows (g/L of dH_2_O): zein (corn protein, 0.3), gluten hydrolysate from corn (0.3), corn starch (0.3), xylan (oat spelt, 0.4), arabinogalactan (larch wood, 2.2), D-lactose (3.2), casein hydrolysate (0.3), whey protein hydrolysate (4.1), peptone from casein (0.5), bactotryptone (0.5), mucin (4.0), yeast extract (standard nucleotide, 2.5), L-cysteine HCl (0.8), 0.05 bile salts, KH_2_PO_4_ (0.5), NaHCO_3_ (1.5), NaCl (4.5), KCl (4.5), MgSO_4_ (1.3), CaCl_2_·2H_2_O (0.1), hemin (0.01), Tween 80 (1.0) and vitamin solution (1 mL/L). The vitamin solution contained (mg/L of dH_2_O): thiamine-HCl (50), riboflavin (50), nicotinic acid (50), pantotenate (100), pyridoxine HCl (100), folic acid (20), cyanocobalamin (5), aminobenzoic acid (50), biotine (20), phylloquinone (0.075), menadione (10). The medium components were dissolved in dH_2_O and the pH adjusted according to the cultivation pH prior to sterilization in an autoclave (20 min at 121°C). The filter-sterilized vitamin solution was added in the medium after sterilization. The cultivation medium used for the first batch fermentation with fecal beads from infant 03 to 09 was a mix of 75% fresh medium and 25% (v/v) of bacteria-free spent medium of a previous continuous fermentation in order to enhance the growth of secondary-metabolite depending species. Short-chain FOS powder (Fibrulose F97, Cosucra, Pecq, Belgium) was dissolved in dH_2_O and sterilized by filtration (0.2 μm) before addition to the sterile cultivation medium at a final concentration of 1, 4 or 8 g/L.

### Experimental set up and sampling

An overview of the experimental set up is shown in Fig. 2. Nine infant fecal microbiota were used for separate immobilization and cultivation in PolyFermS models. Beads produced from one infant fecal microbiota were used to inoculate two or three parallel bioreactors for testing different fermentation factors. The PolyFermS model parameters were adjusted to mimic conditions of the infant colon and fed with medium as presented above. Reactors containing fecal microbiota beads from infant 01 to 03 were supplemented with different concentrations of FOS and operated at pH 5.8 for testing FOS-induced growth of infant-characteristic *Bifidobacterium*. To assess the pH-dependent growth of potential enteropathogens, which are commonly detected in feces of this infant population [5], reactors containing fecal beads from infant 04 to 07 were operated at pH 6.3 or 5.8 with 1 g/L of FOS. Finally, community stability was assessed during long-term cultivation of immobilized fecal microbiota of four Kenyan infants (06 to 09) using the final selected PolyFermS model conditions (1 g FOS/l and pH 5.8) mimicking Kenyan infant microbiota composition and activity.

Effluent samples were collected daily for metabolite analysis with high-performance liquid chromatography (HPLC). Samples were centrifuged for 10 min at 14’000 rpm and 4°C. The supernatant was used for HPLC analysis and the sample pellet was stored at −80°C until further use for DNA extraction.

### Quantitative PCR and 16S rRNA gene amplicon sequencing analysis

The FastDNA SPIN Kit for Soil (MP Biomedicals, Zurich, Switzerland) was used to extract the DNA of fecal (200 mg) and effluent (pellet of 2 mL) samples according to the manufacturer’s instructions.

qPCR was performed to determine the absolute numbers of total bacteria and of selected bacterial targets prevalent in the infant gut microbiota [34] (**Supplementary table 3**). The Roche Light Cycler 480 (Hoffmann-La Roche, Basel, Switzerland) was used as described previously [35]. Reactions were performed in technical triplicates. Each reaction mixture contained 5 μl of SensiFAST SYBR No-ROX mix (Labgene Scientific Instruments, Châtel-Saint-Denis, Switzerland), 0.5 μl of forward and reverse primer (10 μM, Microsynth, Balgach, Switzerland), 3 μl of nuclease free H_2_O and 1 μl of DNA template. A 2-step program was used with 3 min of initial denaturation at 95°C, 40 cycles of 5 seconds at 95°C and 30 seconds at 60°C and a final melting curve analysis from 65 to 97°C at a ramp rate of 0.11°C/s. Standard curves were generated using tenfold dilutions of linearized plasmid containing the gene of interest. Curves showing an amplification efficiency of 80–102% (slope 3.26–3.90) were included for analysis. To obtain the bacterial concentration, qPCR gene copy numbers were adjusted for the median number of 16S rRNA gene copies of each target (Ribosomal RNA Database, **Supplementary table 3**) [36].

The Illumina MiSeq platform was used to perform paired-end 16S rRNA gene amplicon sequencing (Illumina, CA, USA) at the Genetic Diversity Center (GDC, ETH Zürich, Zurich, Switzerland) as previously described [12]. The V4 region of the 16S rRNA gene was amplified with the primer combination nxt_515F/nxt_806R (5′-GTGCCAGCMGCCGCGGTAA-3′, 5′-GGACTACHVGGGTWTCTAAT-3′) followed by amplicon barcoding using Nextera Index primers. Sequencing was performed with the MiSeq reagent kit v2 (2 × 250 bp read length). Removal of Illumina adaptors and gene-specific PCR primers from raw reads was done using Atropos [37]. The DADA2-pipeline [38, 39] was used to generate amplicon sequencing variants (ASV). Forward and reverse reads were truncated after 170 nucleotides and 160 nucleotides, respectively. Truncated reads with an expected error rate higher than three for forward and four for reverse reads were removed. After filtering, denoising, error rate learning and ASV inference, reads were merged with a minimum overlap of 40 bp. Chimeric sequences were removed and taxonomy was assigned using the SILVA database (v.132) [40].

### Metabolite analysis

Main short-chain fatty acids (SCFA, acetate, propionate, butyrate and valerate), branched-chain fatty acids (BCFA, isobutyrate, isovalerate) and intermediate metabolites (succinate, lactate and formate) of fecal and effluent samples were quantified using HPLC as previously described [12]. Total metabolites represent the sum of SCFA, BCFA and intermediate metabolite concentrations. Fecal metabolites were extracted by dissolving 200 mg of feces in 600 μL 10 mM H_2_SO_4_ followed by centrifugation at 6000 g and 4°C for 20 min. Supernatant was filtered through 0.45 μm nylon membranes (Infochroma AG, Zug, Switzerland) into a glass vial and sealed with crimp-caps. A LaChrom HPLC-System (Merck-Hitachi, Tokyo, Japan) was used with a SecurityGuard Cartridge Carbo-H (4 × 3.0 mm; Phenomenex Inc., Torrance, CA, United States) connected to a Rezex ROA-organic acid H + column (300 × 7.8 mm; Phenomenex Inc., Torrance, CA, United States) and an Accela RI detector (Thermo Fisher Scientific Inc., Waltham, MA, United States). Sample volumes of 20 μL were analyzed with a mobile phase of 10 mM H_2_SO_4_ at a flow rate of 0.6 mL/min and a column temperature of 40°C. The data were processed using EZChrom software (Agilent, Santa Clara, CA, United States).

### Data visualisation and statistical analysis

Microbiota community analysis was done in R (version 4.0.4) using the phyloseq [41], vegan [42] and ggplot2 [43] packages. Differential abundance analyses were performed using DESeq2 [44]. Rarefied data were used to calculate relative abundances, alpha and beta diversity. Alpha diversity is a measure of diversity within a community with respect to the number of observed taxa (richness) or abundance distribution of taxa (evenness) [45]. Beta diversity is a measure of similarity or dissimilarity between multiple communities [46]. The binary Jaccard similarity index calculates similarity between communities based on presence/absence of taxa. The weighted Jaccard similarity index additionally considers the abundance of the present taxa. GraphPad Prism (version 9.1.0) was used to create graphs and for statistical analysis. Normal distribution was assessed using the Shapiro-Wilk test. An unpaired t-test was used to test differences between two independent normal-distributed samples and Welch’s correction was applied in case of unequal variances. Wilcoxon rank sum test was carried out for not normal distributed samples. An ordinary one-way ANOVA was applied to test differences between more than two independent normal-distributed samples and in case of statistical significance a Tukey’s post-hoc test was performed. Kruskal-Wallis test was applied for not normal distributed samples with a post hoc Dunn’s test in case of statistical significance. Repeated measures one-way ANOVA was applied to test for differences in alpha diversity and percentage of shared genera over long-term continuous fermentation.

## Figures and Tables

**Table 1:** Characteristics of the fecal donors and measured fecal pH.

Infant	Sex	Age (months)	Feeding	Weaning time (months)	Delivery	Gestation at birth	Birth weight (kg)	Fecal pH
01	Male	7.7	mixed	1.7	Vaginal	na	3.1	5.2
02	Male	5.5	mixed	2.5	Vaginal	na	3.4	4.6
03	Male	9.0	mixed	3.0	Vaginal	Preterm	2.0	5.3
04	Male	7.6	mixed	1.6	Vaginal	Term	3.4	4.3
05	Female	6.1	mixed	0.1	Vaginal	Term	3.2	5.4
06	Male	5.6	mother milk	0.0	Vaginal	Term	2.8	4.8
07	Female	5.6	mother milk	0.0	Vaginal	Term	3.1	5.3
08	Female	8.2	mixed	2.2	Vaginal	Term	2.0	4.8
09	Male	9.7	mixed	4.7	Vaginal	Term	2.0	7.3

mixed: mother milk + complementary foods, na: not available

## Data Availability

The datasets generated during and/or analysed during the current study are available in the European Nucleotide Archive (ENA) repository (https://www.ebi.ac.uk/ena/browser/view/PRJEB63090).
